# Factors impacting knowledge and use of long acting and permanent contraceptive methods by postpartum HIV positive and negative women in Cape Town, South Africa: a cross-sectional study

**DOI:** 10.1186/1471-2458-12-197

**Published:** 2012-03-16

**Authors:** Sarah Credé, Theresa Hoke, Deborah Constant, Mackenzie S Green, Jennifer Moodley, Jane Harries

**Affiliations:** 1 Women's Health Research Unit, University of Cape Town, Cape Town, South Africa; 2 Family Health International 360, North Carolina, USA

**Keywords:** PMTCT, Contraception, Fertility intentions, Unintended pregnancies, HIV, IUD, Female sterilization

## Abstract

**Background:**

The prevention of unintended pregnancies among HIV positive women is a neglected strategy in the fight against HIV/AIDS. Women who want to avoid unintended pregnancies can do this by using a modern contraceptive method. Contraceptive choice, in particular the use of long acting and permanent methods (LAPMs), is poorly understood among HIV-positive women. This study aimed to compare factors that influence women's choice in contraception and women's knowledge and attitudes towards the IUD and female sterilization by HIV-status in a high HIV prevalence setting, Cape Town, South Africa.

**Methods:**

A quantitative cross-sectional survey was conducted using an interviewer-administered questionnaire amongst 265 HIV positive and 273 HIV-negative postpartum women in Cape Town. Contraceptive use, reproductive history and the future fertility intentions of postpartum women were compared using chi-squared tests, Wilcoxon rank-sum and Fisher's exact tests where appropriate. Women's knowledge and attitudes towards long acting and permanent methods as well as factors that influence women's choice in contraception were examined.

**Results:**

The majority of women reported that their most recent pregnancy was unplanned (61.6% HIV positive and 63.2% HIV negative). Current use of contraception was high with no difference by HIV status (89.8% HIV positive and 89% HIV negative). Most women were using short acting methods, primarily the 3-monthly injectable (Depo Provera). Method convenience and health care provider recommendations were found to most commonly influence method choice. A small percentage of women (6.44%) were using long acting and permanent methods, all of whom were using sterilization; however, it was found that poor knowledge regarding LAPMs is likely to be contributing to the poor uptake of these methods.

**Conclusions:**

Improving contraceptive counselling to include LAPM and strengthening services for these methods are warranted in this setting for all women regardless of HIV status. These study results confirm that strategies focusing on increasing users' knowledge about LAPM are needed to encourage uptake of these methods and to meet women's needs for an expanded range of contraceptives which will aid in preventing unintended pregnancies. Given that HIV positive women were found to be more favourable to future use of the IUD it is possible that there may be more uptake of the IUD amongst these women.

## Background

Prevention of mother-to-child transmission (PMTCT) of HIV has been a global priority since the late 1990's [1]. Although great progress has been made in preventing vertical transmission of HIV, nearly half a million children worldwide were newly infected in 2008, of which 90% were as a result of mother-to-child transmission [1]. The World Health Organization (WHO) promotes a four element strategy for PMTCT of HIV: preventing HIV infection among women, preventing unintended pregnancies, preventing perinatal HIV infection through use of antiretroviral prophylaxis, and providing care and support to HIV positive mothers and their families [1]. The prevention of unintended pregnancies among HIV positive women, although highly cost-effective, is a neglected strategy in the fight against HIV/AIDS [2,3].

In South Africa, an estimated 5.2 million people are infected with HIV and women are disproportionately affected [4]. The prevalence of HIV in South Africa has been found to peak among females aged 25-29 years at 32.7% [4]. The Western Cape is the province with the lowest HIV prevalence rates in South Africa although, compared to the rest of the world, the prevalence of HIV (5.3%) among 25-49 year old women [4] is high. In sections of Cape Town with high concentration of low income populations, over 25% of women seeking antenatal care are HIV positive [5]. Thus PMTCT among sexually active HIV positive women is an important component in the fight against HIV/AIDS in this region. Prior research in South Africa and elsewhere in sub-Saharan Africa has shown that many HIV-positive women desire pregnancy, although for some HIV status does diminish aspirations for childbearing [6,7].

Focusing specifically on those HIV positive women who currently want to avoid pregnancy, this intention can be achieved by using a modern contraceptive method. Counselling must be offered in an environment free of coercion and in a way that ensures the client has sufficient knowledge about the full range of contraceptive options to make an informed choice of the method that optimally suits her needs [8]. Evidence is largely lacking, however, on how family planning services can be tailored to meet the needs of HIV-positive women, given that little research has systematically examined how contraceptive knowledge and beliefs vary by sero-status. Studies among HIV positive women have identified male condoms as being the most popular method of contraception, followed by hormonal methods; use of IUDs is rare [9-11]. Method choice has been found to be influenced by previous method use and the need for methods that can be started immediately after delivery [9]. In research conducted in South Africa, one study showed higher contraceptive prevalence among women living with HIV compared to their HIV-negative counterparts [12], while another study conducted with postpartum women showed no such difference [13]. A study with postpartum women in Rwanda found greater contraceptive use among HIV-positive women, along with greater knowledge of modern contraceptive methods [14].

In offering contraceptive services to all women, regardless of sero-status, expanding the range of available methods has been shown to increase both contraceptive prevalence [15,16], and continuation rates [17]. One approach for increasing contraceptive options is to promote and ensure access to long-acting and permanent methods (LAPM) in addition to short-acting methods like condoms, injectables, and oral contraceptives. Long-acting methods like the IUD and implants and permanent methods like male and female sterilization are not dependent on users' adherence. Additionally, continuation does not require repeated contact with health care providers. These two factors make LAPMs more effective than short-acting methods in preventing pregnancies among typical users. Despite high motivation to avoid pregnancy among many HIV-positive women and desire for highly effective contraception, use of LAPMs is typically low [14]. Expanding method choice beyond short acting hormonal methods may become increasingly important in light of new data suggesting a potential link between hormonal contraceptive use and HIV acquisition and transmission [18].

Modern contraceptive methods are available free of charge at public sector health care facilities in South Africa, and use is high: an estimated 65% of sexually active women use a method [19]. The majority of sexually active women in South Africa use short acting methods--primarily injectable contraceptives (33.2%) with 10% of women using female sterilization and less than 1% using the intrauterine device (IUD) [19]. Weak promotion and uptake of LAPMs in South Africa, including among HIV positive women who report that they desire no future pregnancies, has been identified as a gap in service provision [8,13,20].

This cross sectional study examined factors that influenced HIV positive and negative women's choice in contraception, focusing primarily on knowledge and attitudes towards the IUD and female sterilization, the two LAPMs currently available in South African public sector facilities. The study examined whether differences exist between the two populations in terms of factors influencing method choice, with the aim of determining how services could be adapted to respond to the contraceptive needs of HIV positive women.

## Methods

### Study design and setting

The data for this study were derived from a pre-intervention quantitative cross sectional survey undertaken to evaluate knowledge, attitudes, and behaviours related to use of LAPM. Data were collected via face-to-face interviews at four public sector primary care facilities in Khayelitsha and one clinic in Mitchell's Plain in the greater Cape Town area. Selected sites offered both PMTCT and family planning services and had the necessary infrastructure and staffing to offer IUD services. Khayelitsha and Mitchell's Plain are high density areas situated on the outskirts of Cape Town. Poverty and unemployment are high in these areas. Khayelitsha has a very high burden of HIV with 30.1% of women attending antenatal care testing positive [21,22]. Antenatal HIV prevalence in the Mitchell's Plain district is 13.9% [21].

### Sample

Eligible study participants were women seeking child health services for an infant 6 months or younger and who received antenatal care during their most recent pregnancy. Potential participants were identified in the waiting areas at primary health care clinics. Those who were eligible were invited to participate in the order in which they presented at the facility until the intended sample size was achieved. Participants were excluded if they were younger than 18 years of age or could not understand English or Xhosa.

The study aimed to interview 250 women who had used PMTCT services and an equal number of postpartum women who had not used such services, who were presumed to be HIV negative. This sample size, to be repeated post-intervention, provides sufficient power to determine whether the intervention was a success, defined as at least 30% of HIV-positive participants expressing correct knowledge about the IUD and female sterilization as a contraceptive option.

### Data collection

Trained research assistants administered structured questionnaires collecting information on socio-demographic characteristics, contraceptive use, reproductive history and future fertility intentions. The questionnaire also included items on women's exposure to LAPM counselling postpartum and the content of this counselling. Further questions were asked regarding women's knowledge and attitudes towards the IUD and female sterilization as LAPM. Data collection took place from February to May 2009.

### Data analysis

Data were analysed using STATA 10.0 [23]. Summary statistics were estimated separately for HIV positive and HIV negative participants. Descriptive results were compared by HIV status using chi-squared tests or Fisher's exact tests for categorical outcomes and Wilcoxon rank-sum tests for continuous outcomes.

### Ethics

The research was approved by the Health Science Faculty Human Research Ethics Committee of the University of Cape Town (235/2008) as well as by the Western Cape Department of Health (19/18/RP53/2008) and the Protection of Human Subjects Committee of FHI 360. All participants gave written informed consent before enrolment took place.

## Results

### Socio-demographic characteristics

The demographic profiles of the HIV positive and negative participants were similar (Table 1). Participants were most commonly in their mid-twenties, unemployed, had less than a grade 12 education, were unmarried but in a stable relationship and had an average of two living children.

**Table 1:** Summary characteristics, including reproductive history and fertility desires of postpartum HIV positive and negative women attending child health services

*Characteristic*		*HIV Positive n = 265*		*HIV Negative n = 273*		P-value*
		
		*n*	*%*	*n*	*%*	
**Age**	Median (IQR)	27 (23-32)		25 (21-29)		0.144

**Highest level of education†**						0.332

	No formal schooling	0	0.0%	0	0.0%	

	Grade 1-7	19	7.2%	16	5.9%	

	Grade 8-12 without matric	171	64.8%	173	63.4%	

	Grade 12 with matric	66	25.0%	66	24.2%	

	Tertiary qualification	8	3.1%	18	6.6%	

**Current relationship status‡**						0.274

	Married	88	33.3%	93	34.7%	

	Single, stable relationship	166	62.9%	160	59.7%	

	Single, casual relationship	2	0.8%	8	3.0%	

	Single, no relationship	8	3.0%	7	2.6%	

**Employment status**						0.844

	Paid Job (Yes)	66	24.9%	70	25.6%	

	Paid Job (No)	199	75.1%	203	74.4%	

**Number of living children**	Median (IQR)	2(1-2)		2(1-2)		0.632

**Time since delivery (months)**	Median (IQR)	2.20 (1.28-3.43)		2.40 (1.48-3.38)		0.049

**Last pregnancy intended§**						0.696

	Yes	101	38.4%	100	36.8%	

	No	162	61.6%	172	63.2%	

**Would like another child**						< 0.001

	Yes	30	11.3%	74	27.1	

	No	194	73.2%	168	61.5%	

	Unsure	41	15.5%	31	11.4%	

		n = 30		n = 74		

**Desired timing of next child**^**ǁ**^						0.684

	< 12 months	0	0%	0	0%	

	12-24 months	1	3.3%	3	4.2%	

	25-36 months	3	10%	6	8.3%	

	> 36 months	13	43.3%	40	55.6%	

	Unsure	13	43.3%	23	31.9%	

Missing values excluded from denominators in calculation of percentages.

*P-values for comparisons using Wilcoxon sum-rank and chi-squared tests

†One missing value in HIV+

‡Two missing values in HIV + and five missing values in HIV-,

§ Two missing values in HIV + and one missing value in HIV-

**^ǁ^**Two missing values in HIV-

### Pregnancy history and fertility desires

The majority of women in both samples reported their most recent pregnancy was unintended (not planned) (Table 1). Significantly more (p < 0.001) HIV positive women either did not want another child or were unsure whether they wanted another child. Of all the women who expressed interest in having another child, the vast majority wanted to wait at least three years or were unsure of when they wanted their next child.

### Current use of contraception

Nearly ninety per cent of HIV positive and HIV negative participants reported that they were currently using a modern method of contraception (Table 2). The vast majority of women in both samples were using short acting methods, primarily the 3-monthly injectable, Depo Provera (DMPA). No IUD use was reported, and a similarly small percentage of participants reported they had undergone female sterilization. A small percentage of women (3.8% HIV positive and 1.7% HIV negative) were using condoms in addition to a hormonal method of contraception or sterilization (data not shown).

**Table 2:** Use of contraception and most common reasons reported for method choice by type of contraceptive method currently using

	HIV Positive n = 265		HIV Negative n = 273		P-value*
	**n**	**(%)**	**n**	**(%)**	

**Currently using a family planning method**					0.763

Yes	238	89.8%	243	89.0%	

No	27	10.2%	30	11.0%	

	**n = 238**		**n = 243**		

**Method currently used**					

IUD	0	0%	0	0%	N/A

Male Sterilization	0	0%	1	0.4%	N/A

Female Sterilization	17	7.1%	14	5.8%	0.537

Condoms†	13	5.5%	6	2.5%	0.105

Pills	2	0.8%	6	2.5%	0.285

2 monthly injectable	48	20.2%	59	24.3%	0.278

3 monthly injectable	167	70.2%	160	65.8%	0.309

	**Hormonal methods** **(Injectables and Pills)**				

	**HIV Positive** **n = 217**		**HIV Negative** **n = 225**		

**Reason for hormonal method choice§**					

Method is convenient for me	110	50.7%	99	44%	0.159

Provider recommended the method	106	48.9%	133	59.1%	0.030

No side effects	18	8.3%	30	13.3%	0.089

Effective in preventing pregnancy	12	5.5%	25	11.1%	0.034

Partner approves of method	0	0%	1	0.5%	N/A

	**Female sterilization**				

	**HIV Positive** **n = 17**		**HIV Negative** **n = 14**		

**Reason for female sterilization method choice§**					

Method is convenient for me	15	88.2%	10	71.4%	0.370

Effective in preventing pregnancy	7	41.2%	10	71.4%	0.149

Provider recommended the method	5	29.4%	2	14.3%	0.412

Partner approves of method	4	23.5%	4	28.6%	1.00

No side effects	0	0%	2	14.3%	0.196

*P-values for bi-variate comparisons using Chi-squared test or Fisher's exact test

†Includes use of male and female condoms

§Respondents could name more than one reason; this table presents the four most common reasons for each method group.

Table 2 presents participants' reasons for contraceptive choice, stratified by HIV status and method category (hormonal versus sterilization; no IUD use was reported). The four most common reasons given for hormonal method choice were: convenience, provider recommendation, absence of side effects and method effectiveness in preventing pregnancy. While these reasons were most commonly mentioned in both samples, significantly more HIV negative women (p = 0.034) mentioned that their method choice was based on its effectiveness in preventing pregnancy. Furthermore 'provider recommended the method' was significantly different for hormonal method choice, with a greater percentage of HIV negative women stating this as a reason. The most commonly mentioned reason for choice of female sterilization in both samples was convenience; most HIV-negative women also mentioned high effectiveness as a factor influencing choice of sterilization. While there were no significant differences between the two samples for their choice of female sterilization notable percentage differences can be seen. A greater percentage of HIV negative women stated that their reason for choosing female sterilization was due to its effectiveness in preventing pregnancy, while more than double the number of HIV positive women mentioned that their choice was based on provider recommendation.

### Exposure to counselling and attitudes and knowledge regarding LAPM

Participants were asked whether a provider had talked to them about family planning methods in the time since last becoming pregnant. A greater proportion of HIV negative women reported that a provider had discussed family planning methods, with just under half of HIV positive women reporting exposure to such messages (Table 3). Similarly few women in both samples reported being told about the IUD. More participants reported that a provider had told them about female sterilization since last becoming pregnant, with no difference between the two groups. Most commonly this information was presented to participants in a group (51.2%- data not shown in table).

**Table 3:** Exposure to IUD or sterilization counselling and attitudes among women unwilling to try the method in the future†

	HIV Positive	HIV Negative	P-value*
	
	n (%)	n (%)	
	**n = 265**	**n = 273**	

**Counselled by provider on family planning methods**			

Yes	128(48.3%)	160(58.6%)	0.017

No	137(51.7%)	113(41.4%)	

	**n = 128**	**n = 160**	

**Counselled by provider on IUD †**			

Yes	10(7.8%)	16(10.0%)	0.520

No	118(92.2%)	144(90.0%)	

**Overall opinion of IUD**			

Favourable	210(79.3%)	188(68.9%)	0.001

Unfavourable	34(12.8%)	70(25.6%)	

No opinion	21(7.9%)	15(5.5%)	

	**n = 44**	**n = 52**	

**Reasons for not using IUD in future ‡**			

Prefer sterilization/already sterilized	11(25.0%)	13(25.0%)	1.00

Concern about/Fear of Insertion and removal procedure	9(20.5%)	11(21.2%)	0.933

Not sure if safe with current health status	9(20.5%)	2(3.9%)	0.021

More info needed	5(11.4%)	4(7.7%)	0.728

Don't know enough or scared	4(9.1%)	4(7.7%)	1.00

Unsure	5(11.4%)	8(15.4%)	0.766

	**n = 128**	**n = 160**	

**Counselled by provider on female sterilization†**			

Yes	74(57.8%)	74(46.3%)	0.051

No	54(42.2%)	86(53.8%)	

**Overall opinion of Sterilization**			

Favourable	201(75.9%)	196(71.8%)	0.561

Unfavourable	43(16.2%)	51(18.7%)	

No opinion	21(7.9%)	26(9.5%)	

	**n = 57**	**n = 80**	

**Reasons for not using sterilization in future §**			

Permanent	30(52.6%)	57(71.3%)	0.026

Surgical procedure	10(17.5%)	22(27.5%)	0.175

Too young/not married	18(31.6%)	25(31.3%)	0.967

Need more information	3 (5.3%)	16(20.0%)	0.014

Afraid of procedure	13(22.8%)	16(20.0%)	0.692

No protection against STIs and HIV	3(5.3%)	3(3.8%)	0.693

*P-values for comparisons using Chi-squared and Fisher's exact

†Denominator is women who received family planning counselling

‡Participants could report several reasons; those shown where n > 5. Denominator is women not willing to try the IUD in the future

§Participants could report several reasons; those shown where n > 5. Denominator is women not willing to try female sterilization in the future.

The number of women reporting that they were in favour of the IUD, when asked whether their overall opinion about the IUD was favourable or unfavourable, was significantly greater (p = 0.001) among HIV positive (79.3%) than HIV negative (68.9%) participants. Reasons for not wanting to use the IUD among women unwilling to try the method in the future are presented in Table 3. Significantly more (p = 0.021) HIV positive women reported that they would not use the IUD in future as they were not sure if it was safe to use with their current health status (20.5% HIV positive vs. 3.9% HIV negative). In contrast, participants' overall opinion of sterilization was similar and favourable for 75.9% of HIV positive participants and 71.8% of HIV negative participants (Table 3). Looking at the reasons for not using sterilization in the future, significantly more HIV negative women stated that method permanence and lack of information about the method were their reasons for not wanting to use sterilization. For both the IUD and female sterilization concerns regarding the procedures involved were identified as reasons why women do not want to use these methods in the future (Table 3).

When asked how an HIV positive woman can avoid transmitting HIV to her infant, the most common answer among both HIV positive and negative women was by attending PMTCT services offering antiretroviral prophylaxis for the woman and the newborn (86.4% HIV positive and 72.2% HIV negative) (Data not shown). In addition significantly more HIV negative women (30.0% versus 21.5%, p = 0.002) reported that avoiding pregnancy would aid in the prevention of mother to child transmission.

**Figure 1:**
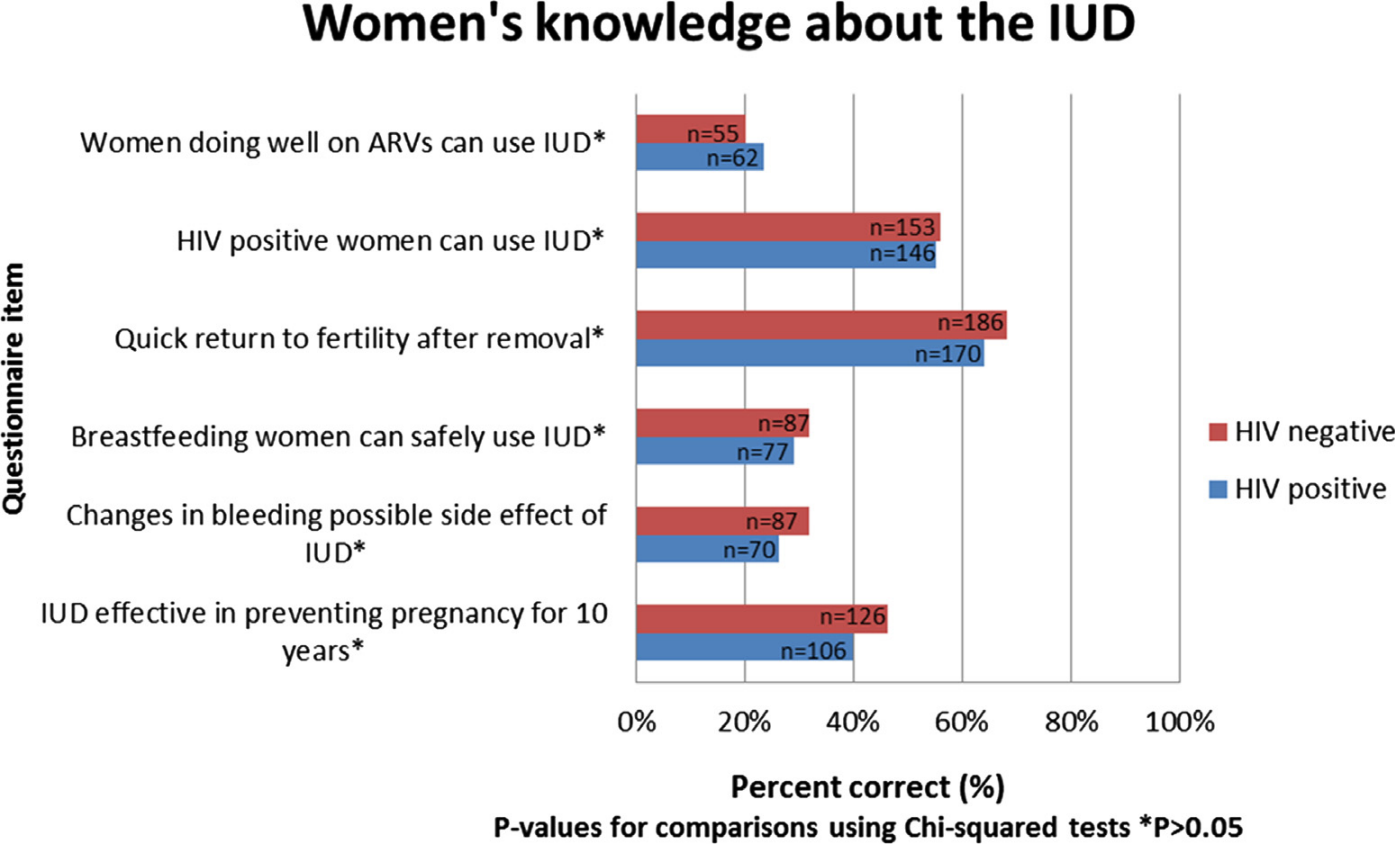
**Women's knowledge about the IUD**.

In response to the six questions asked to determine women's knowledge about the IUD as a LAPM, more than half of all participants, both HIV positive and negative, either did not know or were unsure that: women doing well on antiretrovirals (ARVs) can use an IUD; that breastfeeding women can use the IUD; that changes in bleeding patterns are a possible side effect of the IUD or that the IUD is effective in preventing pregnancy for 10 years (Figure 1).

**Figure 2:**
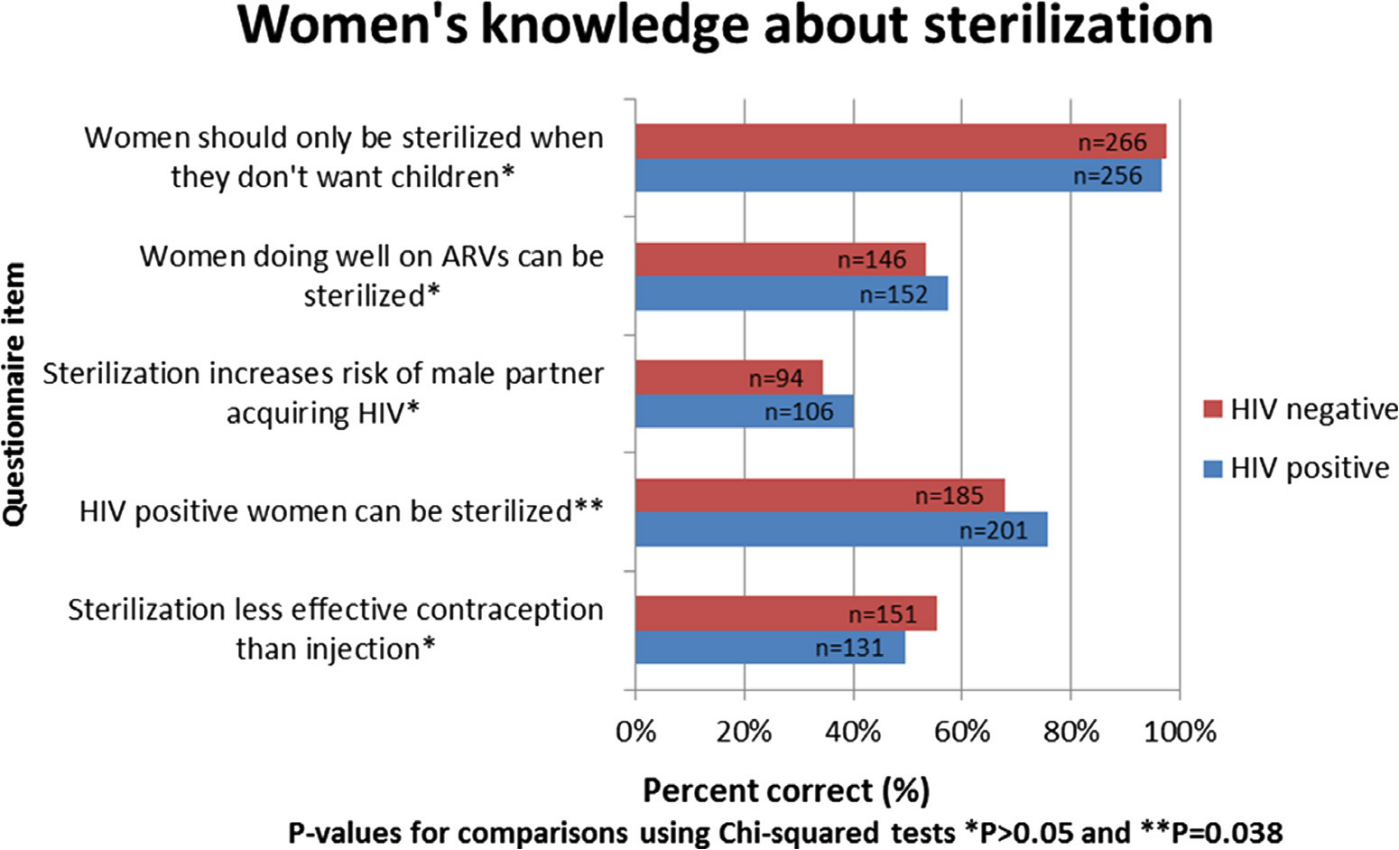
**Women's knowledge about sterilization**.

Five questions were asked to ascertain women's knowledge about sterilization. More than 90% of women in both samples knew that sterilization should only be used by women who do not want to have more children (Figure 2). Half the HIV negative participants and 44.1% of HIV positive participants did not know or were unsure that sterilization is a more effective method of contraception than the injection.

## Discussion

This study documented low use of the IUD and female sterilization among postpartum women seeking child health services for their infants in public sector facilities, with negligible difference based on HIV status. This finding is not unexpected, in that data collection was conducted prior to an intervention to strengthen promotion and provision of an expanded range of contraceptive options, including long acting and permanent methods. More surprising was the finding that the large majority of interviewed women were already using injectable contraceptives, despite being on average just over 2 months postpartum. High reliance on injectables likely reflects the practice of some of South Africa's public sector facilities to offer injectables immediately post-delivery; it also mirrors contraceptive method patterns among South African women in general [19].

The study results suggest high potential demand for highly effective, long acting and permanent methods of family planning that enable women to space their children or to avoid pregnancy entirely, according to their desires. Most women interviewed reported that their most recent pregnancy was not planned. Amongst the HIV positive women nearly three quarters wanted to avoid a future pregnancy. Of interview participants who did want a future pregnancy, more than 80% wanted to wait at least 36 months before their next pregnancy. For some women who have achieved their desired family size or who are highly motivated to avoid pregnancy, injectable contraceptives may not be the ideal method choice. Method effectiveness depends on the client making timely visits to a health service provider four to six times per year for re-injection; women's success with the method also depends on facilities maintaining consistent supplies. Prior research in South Africa has revealed the substantial number of injectable users who return late for re-injections, leading some to suspend at least temporarily use of any method [24].

This study revealed knowledge gaps and biases that must be addressed in efforts to increase use of LAPM. The results show a substantial gap between the proportion of women saying they are in favour of LAPM and the proportion actually using them. Even though significantly more HIV positive women were in favour of the IUD, among women saying they would not use the IUD in the future, significantly more HIV positive women stated as a reason that they were not sure it was safe given their health status.

Of importance, particularly in high HIV prevalence settings, is that the IUD can be used on clinically well HIV positive women [25]. This message needs to be communicated to all participants during contraceptive counselling. In addition, many women did not want to use LAPM in the future due to concerns about the procedures involved and the fear associated with these procedures. Women's fear of invasive procedures poses a challenge in encouraging the uptake of the IUD and sterilization. However, most women interviewed did not have sufficient knowledge about LAPM to make a fully informed method choice. Participant knowledge of sterilisation was greater than that regarding IUDs in this study, probably owing to the presence of sterilisation services in the Western Cape and the lack of IUD promotion and use. Yet for both the IUD and sterilization, "needing more information" was a reason given for not intending to use these LAPMs in the future. The lack of knowledge regarding the IUD, in particular, is evident where question after question were answered incorrectly or women stated that they were unsure about the correct answer. This suggests that counselling on the full range of available contraceptives was ineffective at the time of the survey.

The permanence of female sterilization was identified by 63.5% of interviewed women as a reason for not wanting to undergo the procedure, reasoning that must be fully respected. Other long acting methods such as the IUD should be offered as a possible alternative. The IUD is a method that is more than 98% effective in preventing pregnancy [26] and allows a quick return to fertility after removal thus allowing women to plan and space their children effectively. A woman using the IUD is not required to attend repeat follow up visits at the clinic and thus this method can be seen as convenient. Among the most commonly stated reasons for current use of hormonal methods, among the study sample, was that the method was convenient. Thus the convenience of LAPM should be communicated to women during counselling if this is a determining factor in their choice of method. It can be seen that the IUD provides a highly effective and convenient method to HIV-positive and -negative women wanting to plan their families and space their children. To maximize service effectiveness, family planning services must capitalize more on the safe, effective technologies that already exist but which are not easily accessible to participants.

Our findings revealed the important influence that health care providers have over women's use of contraceptive methods. Only about half of all participants reported having been told by a provider about contraceptive methods since last becoming pregnant, HIV positive women being even less likely to be so informed. These results raise questions as to whether the recommended education regarding healthy timing and spacing of children is being adequately addressed in postpartum health care services and whether women are given sufficient method choice. Furthermore as only a small fraction of participants were informed about the IUD and 52% overall were counselled on sterilization it is questionable whether the full range of contraceptive methods are available and promoted in all services targeting pregnant and postpartum women. Meanwhile, 55.1% of injectable users stated that a reason for method choice was because a provider recommended it. This finding is consistent with other studies where provider related factors influenced method choice [27]. Women in all communities should have access to the full range of methods available in the public health care setting to enable them to choose the most suitable method and to promote consistent and correct use. If women do not have access to the full range of available contraceptive methods this may result in women using methods that are known to have relatively high failure rates amongst typical users [27]. Through expanding contraceptive options the gap between women's reported childbearing intentions and actual childbearing would be able to be narrowed [28].

## Conclusions

The high rates of unintended pregnancies in the sample of HIV positive women suggest that the WHO's strategy of preventing unintended pregnancies amongst HIV positive women to minimise vertical transmission of HIV must be reinforced. Long acting and permanent methods could fill an important gap in family planning services in South Africa given women's stated fertility preferences indicating a strong desire to either not have a future pregnancy or to wait several years before the birth of their next child. Given the high contraceptive prevalence recorded in the target population, half of whom were PMTCT participants, the challenge in providing services lies not in encouraging timely postpartum family planning uptake; rather, it lies in encouraging consideration of an expanded range of contraceptive options, including LAPM.

Future efforts must focus on increasing women's knowledge about safe, effective and long-acting contraceptive options, thereby preparing them to make a fully informed method choice. This can be achieved by strengthening providers' capacity to promote and deliver an expanded range of contraceptive methods and establishing provision of such comprehensive services as a standard performance expectation. The similar rates of unintended pregnancies, similar reasons for contraceptive method choice and similar knowledge and attitudes about LAPM between HIV positive and negative women highlight the need for improved family planning counselling for all women. Initiatives focused on serving the postpartum family planning needs of PMTCT participants should focus first on resolving challenges associated with facilitating uptake of highly effective and permanent contraceptive methods by all women, regardless of HIV status.

## Competing interests

The authors declare that they have no competing interests.

## Authors' contributions

All authors participated in the design of the study. SC co-ordinated the study. SC and TH wrote the first draft of the manuscript. SC, DC and MG performed the statistical analysis. All authors contributed to the writing of this paper and read and approved the final manuscript.

## Pre-publication history

The pre-publication history for this paper can be accessed here:

http://www.biomedcentral.com/1471-2458/12/197/prepub
